# Harnessing flow-induced vibrations for energy harvesting: Experimental and numerical insights using piezoelectric transducer

**DOI:** 10.1371/journal.pone.0304489

**Published:** 2024-06-10

**Authors:** Md Islam, Ussama Ali, Shital Mone

**Affiliations:** 1 Mechanical Engineering Department, Khalifa University of Science and Technology, Abu Dhabi, UAE; 2 Mechanical Engineering Department, University of Engineering and Technology, Lahore, Pakistan; NED University of Engineering and Technology, PAKISTAN

## Abstract

Flow-induced vibrations (FIV) were considered as unwanted vibrations analogous to noise. However, in a recent trend, the energy of these vibrations can be harvested and converted to electrical power. In this study, the potential of FIV as a source of renewable energy is highlighted through experimental and numerical analyses. The experimental study was conducted on an elastically mounted circular cylinder using helical and leaf springs in the wind tunnel. The Reynolds number (*Re*) varied between 2300–16000. The motion of the cylinder was restricted in all directions except the transverse direction. The micro-electromechanical system (MEMS) was mounted on the leaf spring to harvest the mechanical energy. Numerical simulations were also performed with SST *k*–*ω* turbulence model to supplement the experiments and were found to be in good agreement with the experimental results. The flow separation and vortex shedding induce aerodynamic forces in the cylinder causing it to vibrate. 2S vortex shedding pattern was observed in all of the cases in this study. The maximum dimensionless amplitude of vibration (*A/D*) obtained was 0.084 and 0.068 experimentally and numerically, respectively. The results showed that the region of interest is the lock-in region where maximum amplitude of vibration is observed and, therefore, the maximum power output. The piezoelectric voltage and power output were recorded for different reduced velocities (*Ur* = 1–10) at different resistance values in the circuit. It was observed that as the amplitude of oscillation of the cylinder increases, the voltage and power output of the MEMS increases due to high strain in piezoelectric transducer. The maximum output voltage of 0.6V was observed at *Ur* = 4.95 for an open circuit, i.e., for a circuit with the resistance value of infinity. As the resistance value reduced, a drop in voltage output was observed. Maximum power of 10.5μW was recorded at *Ur* = 4.95 for a circuit resistance of 100Ω.

## 1. Introduction

The global energy demand keeps on rising every year [1]. Energy production via conventional sources has adverse effects on the environment, such as global warming and depletion of nonrenewable resources [2]. Over the past two decades, there has been a shift towards renewable energy sources for energy production. One of the United Nations (UN) Sustainable Development Goals (SDG) is “Affordable and Clean Energy” (Goal 7), which stresses the need to produce energy via green and renewable sources [3].

This paper is focused on producing clean energy using flow-induced vibration (FIV) of a constrained structure. FIV refers to the vibration resulting in a structure due to the fluid flow [4]. Generally, any structure might experience FIV if certain conditions are met. FIV is commonly experienced in offshore structures, bridge towers and cables, tall stacks, and pipelines [5]. FIV can result in significant amplitude vibrations if the vibrational frequency (*f*_*vib*_) synchronizes with the natural frequency (*f*_*n*_) of the structure, a condition called “lock-in” [6]. These vibrations are considered detrimental to the structure as they can cause severe damage or complete failure of the system. FIV is governed by various parameters, such as Reynolds number (*Re*), reduced velocity (*Ur*), structure shape, structural damping factor (*C*) and stiffness (*K*), etc. [7].

The mechanism behind the FIV is the detachment of shear layers from the lateral sides of the body which roll up in the wake to form vortices [8]. Boundary layer separation occurs when a layer of fluid near the surface of a structure becomes detached from the surface, leading to the formation of vortices and turbulence in the flow. This induces unsteady aerodynamic forces in the body leading to the FIV [9]. Boundary layer separation and vortex shedding can cause significant flow-induced vibrations in structures and components, such as pipelines, bridges, towers, and more [10]. Although the FIV were generally considered as unwanted and harmful vibrations, recent research is being done to harvest the energy of these vibrations by converting the mechanical energy to electrical energy [11,12]. The two types of FIV, based on the direction of motion, are transverse FIV [13] and streamwise FIV [14]. However, as the transverse vibrations are more prominent than their counterpart, for energy harvesting purposes, only transverse FIV are considered. The region of lock-in is of the most interest as the vibrational energy is maximum in this region which can be converted to electrical energy [15,16].

Energy harvesting from flow-induced vibrations is a promising technology for transforming kinetic energy from fluid flow into electrical energy [17]. The ability to generate power from the energy of fluid flow has the potential to revolutionize renewable energy and contribute to the global effort to combat climate change. The most common examples of energy harvesting from fluid flow are wind turbines, hydroelectric power plants, tidal energy, ocean thermal energy conversion, wave energy, and river current energy, etc. [18]. One specific method of harvesting energy from flow-induced vibrations (FIV) is through the use of piezoelectric devices [19,20]. Piezoelectric transducers are capable of converting mechanical strain into electrical energy and vice versa. This property makes them ideal for harvesting energy from flow-induced vibrations, where the vibration and deformation of a piezoelectric material is caused by the flow of a fluid. Energy harvesting from FIV aligns with the United Nations Sustainable Development Goals, particularly Goal 7, which seeks to “ensure access to affordable, reliable, sustainable, and modern energy for all by 2030” [3,21]. By harnessing the energy of fluid flow, this technology can provide a clean and renewable source of energy that does not produce harmful greenhouse gas emissions. Moreover, the widespread adoption of this technology can help mitigate the effects of global warming, which is primarily caused by the emission of greenhouse gases from traditional energy sources such as coal, oil, and gas [22,23]. By reducing the reliance on these fossil fuels, energy harvesting from FIV has the potential to significantly reduce greenhouse gas emissions and contribute to a sustainable future.

There has been a growing body of research in recent years focused on energy harvesting from flow-induced vibrations. Some of the prominent research methods of harvesting energy from FIV include:

- Energy harvesting from flow-induced vibrations in flexible structures, such as cantilever beams and plates. These structures are designed to vibrate in response to fluid flow, with the vibrations converted into electrical energy using piezoelectric materials or electromagnetic generators [24].
- Energy harvesting from vortex- and wake-induced vibrations in bluff bodies, such as cylinders and spheres, exposed to fluid flow [25,26].
- Hybrid energy harvesting systems that combine multiple energy harvesting techniques, such as piezoelectric materials and electromagnetic generators, to optimize the energy conversion efficiency [27].

A few of the prominent works on energy harvesting from FIV using piezoelectric transducers solely are presented here. Modir and Goudarzi [28] experimentally investigated the influence of spring stiffness on energy harvesting from FIV under high *Re* (1.5×10^4^ ≤ *Re* ≤ 6×10^4^). Their results revealed that the spring stiffness affected the maximum vibrational amplitude and the lock-in range. They also reported that by increasing *f*_*n*_ of the system, the response amplitude increased and so did the amount of harvested energy. Mehdipour et al. [29] used flexible piezoelectric flags attached to the cylinder to harvest the FIV energy. They studied the effect of cylinder cross-sectional shape on the performance of the energy harvesting setup. Their study involved numerical analysis to evaluate lift and drag on the bluff bodies, and experimental apparatus was used to harvest energy. They reported that at low wind speeds the bluff body with a higher drag coefficient generated more power. Table 1 summarizes the recent studies focused on piezoelectric energy harvesting from FIV. The limited amount of work stresses the fact that more research should be carried out in this area.

**Table 1:** Summary of previous research on energy harvesting from flow-induced vibrations.

Authors	Wind tunnel test section (mm)	FIV type	Piezoelectric material (mm)	Max. velocity (m/s)	Max. output (mW)
Kwon (2010) [30]	-	Galloping	PZT (5A M-2814-P2) *l×w×h* = 28 × 14 × 0.2	4	4
Alhadidi & Daqaq (2016) [31]	*L×W×H* = 305 × 305 × 610	WIV	MFC *l×w×h* = 95 × 12.5 × 0.2	6	5
Hu et al. (2018) [32]	*D*_*X*_ = 120	WIV	PZT *l×w×h* = 70 × 20 × 0.25	0.75	0.37
Wang et al. (2019) [33]	*D*_*X*_ = 400	VIV	MFC (2807-P2) *l×w×h* = 37 × 11 × 0.3	2	1.2
Liu et al. (2020) [34]	-	VIV	MFC (M2807-P2) *l×w×h* = 37 × 11 × 0.3	10	1.2
Elahi et al. (2020) [35]	*D*_*X*_ = 900	Flutter	PZT *l×w×h* = 66 × 37 × 0.5	40	6.72
Wang et al. (2021) [36]	*D*_*X*_ = 70	VIV	PZT (NC-PWEH) *l×w×h* = 80 ×40 ×0.2	40	2.87
Wang et al. (2021) [37]	*D*_*X*_ = 400	Galloping	PZT (PZT-5) *l×w×h* = 30 × 20 × 0.5	5	143.6
Hu et al. (2021) [38]	*D*_*X*_ = 400	Galloping	PZT *l×w×h* = 28 × 14 × 0.3	3	0.12
Agarwal & Purohit (2023) [39]	*W×H* = 40 × 40	Flutter	MFC (M8514-P2) *l×w×h* = 100 × 18	5	1.16

PZT = Lead Zirconium Titanate, MFC = Macro-Fiber Composite, *L×W×H* = length × width × height of the wind tunnel test section, *D*_*X*_ = diameter of the cross-section of the wind tunnel test section, *l×w×h* = length × width × height of the piezoelectric strip, VIV = vortex-induced vibrations, WIV = wake-induced vibrations.

This present study explores the transformative potential of flow-induced vibrations as a source of renewable energy by harnessing transverse vibrations through a piezoelectric generator. Highlighting a significant shift from mitigating FIV to actively harvesting energy, the research outlines a detailed experimental setup using an elastically mounted circular cylinder and a micro-electromechanical system (MEMS). Furthermore, the experimental findings are complemented with numerical validation and analysis to provide deeper understanding. The findings demonstrate the possibility of converting these vibrations into electrical energy, aligning with global sustainable development goals by offering a method for generating clean energy from environmental forces like wind and water flows. This approach not only offers green energy but also contributes to mitigating global warming, setting a precedent for further research in piezoelectric energy harvesting and offering practical insights for engineers in the field. The rest of the manuscript is structured as follows: Section 2 presents the experimental setup and the numerical methodology carried out to perform the analysis. The results obtained through the experimental and numerical analysis are discussed in Section 3. Finally, the conclusions drawn from this study are presented in Section 4.

## 2. Methodology

This section provides the description of the experimental setup, procedures, and the numerical method used.

### 2.1 Experimental setup

**Fig 1:**
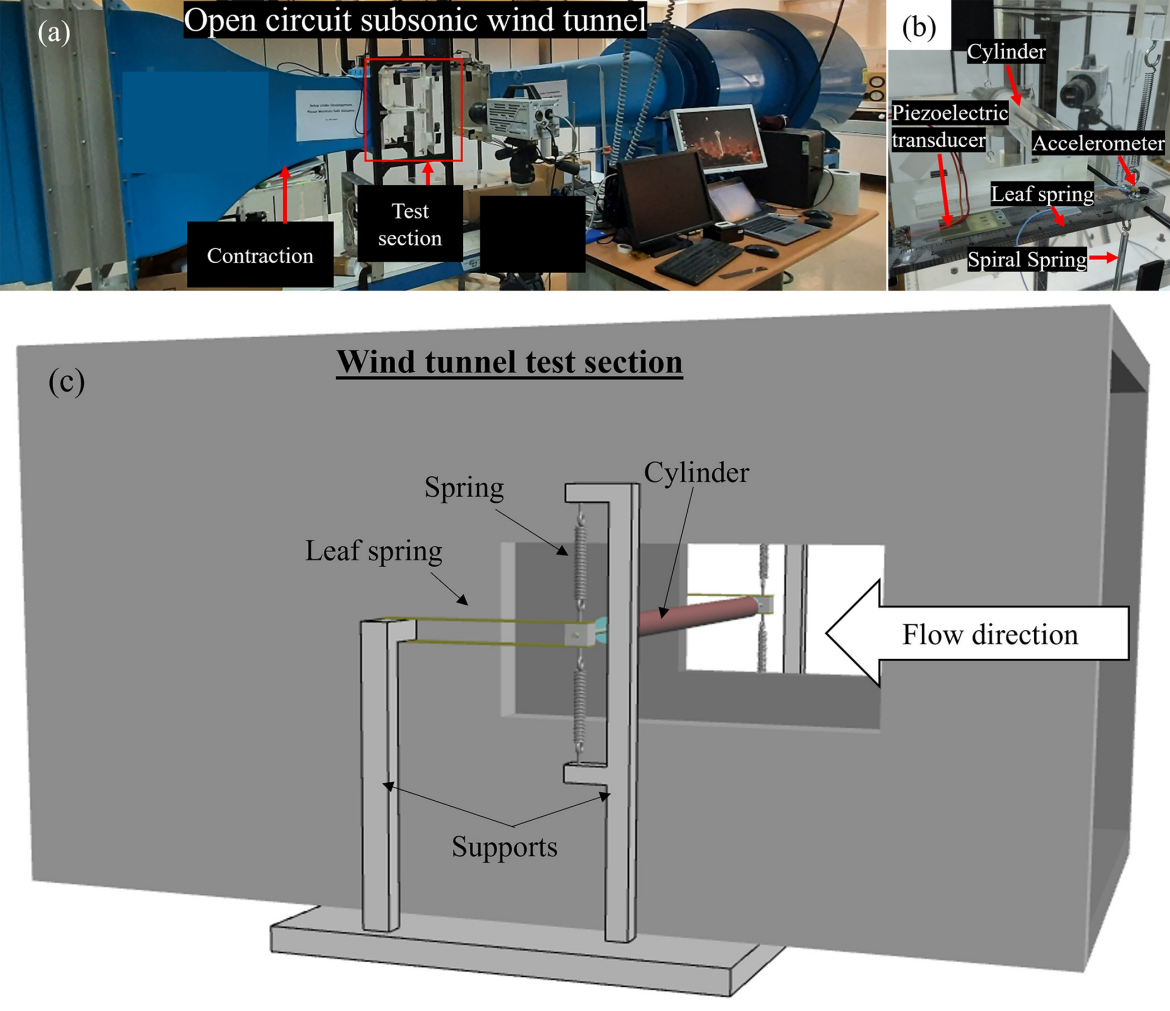
(a) Open circuit subsonic wind tunnel, (b) Close-up of the test section, and (c) 3D model of the wind tunnel test section with elastically mounted cylinder.

**Fig 2:**
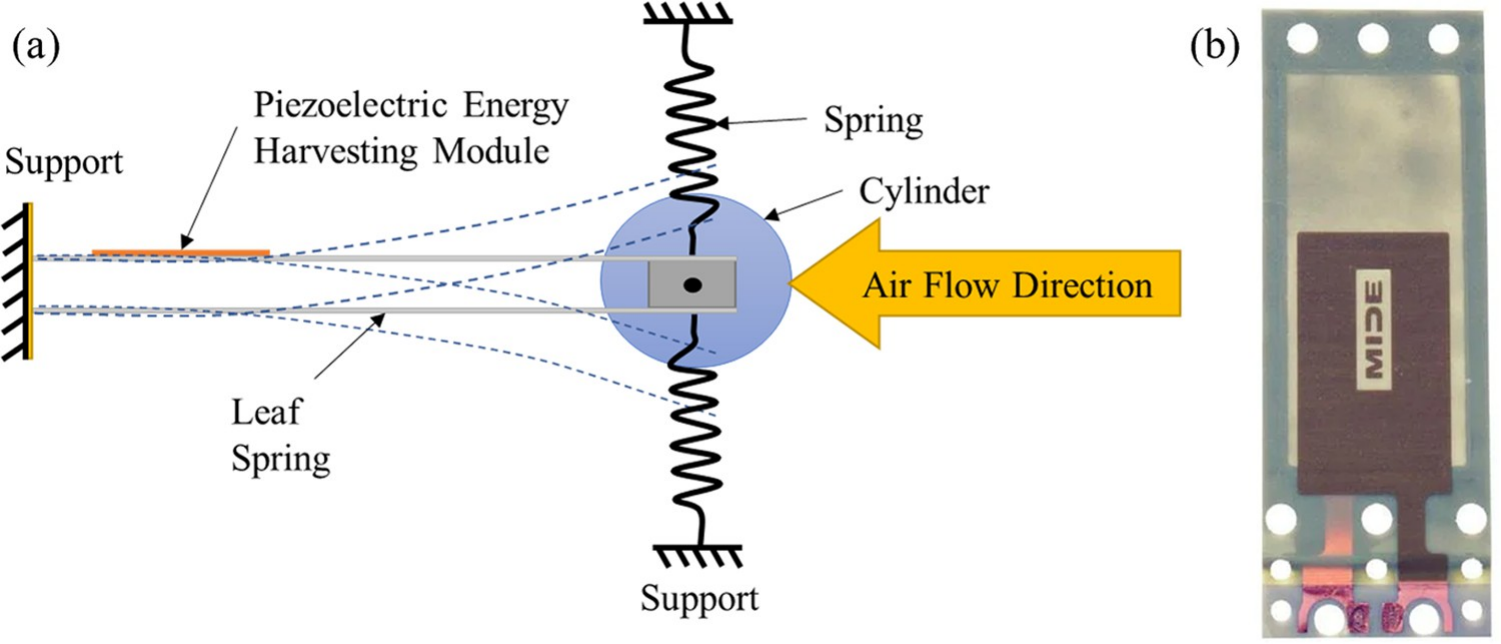
(a) Simple schematic of elastically mounted cylinder with the position of piezoelectric transducer, and (b) actual image of the piezoelectric transducer used in this study.

The experimental setup consisted of a low-speed open-circuit wind tunnel with a test section of 300×300mm cross-section with 600mm length. An elastically mounted acrylic cylinder with an outer diameter (*D*) of 50 mm was subjected to airflow inside the wind tunnel’s test section as shown in Fig 1. The cylinder was supported using two leaf springs and four helical springs on both sides of the cylinder as shown in Figs 1 and 2A. This arrangement allowed the cylinder to oscillate in transverse (*y*) direction only. The micro-electromechanical system MEMS (Mide S230-J1FR-1808XB) shown in Fig 2B, which is a piezoelectric bending transducer, was installed on the leaf spring. It has dimensions of 71.1×25.4 mm and thickness of 0.76 mm. Further details about the transducer can be found in Ref. [40]. Due to the flow of the air over the cylinder, the cylinder started to oscillate once the critical Reynolds number was surpassed. These mechanical oscillations were passed to the MEMS device through the leaf spring which caused bending in the MEMS. The piezoelectric transducer converted this bending strain into electrical energy. The output voltage (*V*) and the oscillation amplitude (*A*) of the cylinder for different reduced velocity (*Ur*) values were measured and recorded by National Instrument’s (NI) Data Acquisition system (DAQ) using a computer.

**Fig 3:**
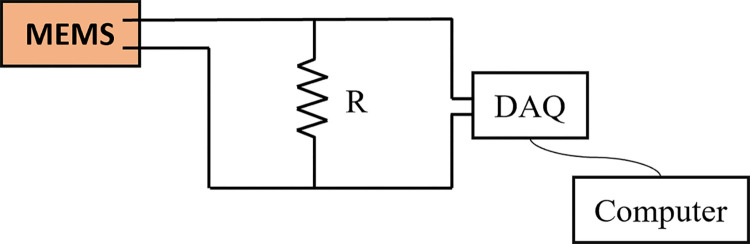
MEMS and resistance connections with DAQ.

The experiment was conducted for an open circuit and with a load by placing the resistance in the MEMS and DAQ circuit, as shown in Fig 3. For the open circuit case, the circuit was completed without placing the resistance in the circuit. With the resistance cases, the resistance values used were 7.5MΩ, 0.69MΩ, 15kΩ, and 100Ω.

The basic important parameters given in Table 2 were calculated before the experiment started. This provided information related to the whole mounting system. The mass of the cylinder was recorded using an electronic scale. The cylinder’s volume was then calculated, enabling the computation of its mass ratio (m* = m/m_*f*_), which is the ratio of the cylinder mass (*m*) to the mass of the fluid displaced (*m*_*f*_), as given in Eq 1. Before conducting wind tunnel tests, the equivalent spring constant (*K*_*eq*_) for the spring-mass system was established by hanging weights from the cylinder and observing the resulting displacement. The system’s natural frequency (*f*_*n*_) and the average logarithmic decrement (*δ*) were estimated from the acceleration versus time plot and power spectrum plot of the cylinder measured using an accelerometer sensor when the cylinder was plucked in the still air (further explained in Section 3). A Fast Fourier Transform (FFT) of the acceleration signal indicated the natural frequency of 9.17 Hz. Additionally, the average logarithmic decrement (*δ*) was computed using Eq 2, which involves the amplitudes of consecutive peaks (*x*_*1*_ and *x*_*2*_) in the acceleration signal. The damping ratio (*ζ*) was then calculated using Eq 3. Equivalent damping factor (*C*_*eq*_) was calculated using Eq 4. The reduced velocity (*Ur*) was adjusted by changing the incoming flow speed (*U*_*∞*_), as outlined in Eq 5, which also altered the Reynolds number (*Re*), according to Eq 6.


$$m^{*}=\frac{m}{m_{f}}=\frac{4m}{\pi \rho D^{2}l_{c}}$$ (1)

$$\delta =\ln(\frac{x_{1}}{x_{2}})$$ (2)

$$\zeta =\frac{\delta /2\pi }{\sqrt{1+{\left(\delta /2\pi \right)}^{2}}}$$ (3)

$$\zeta =\frac{C_{eq}}{2\sqrt{K_{eq}m}}$$ (4)

$$Ur=\frac{U_{\infty }}{f_{n}D}$$ (5)

$$Re=\frac{\rho U_{\infty }D}{\mu }$$ (6)

Where, *D* and *l*_*c*_ are the cylinder diameter and length, respectively, and *ρ* and *μ* are the density and dynamic viscosity of air, respectively.

**Table 2:** Experimental setup structural parameters.

Parameter	Symbol	Value	Unit
Mass of the cylinder	*m*	0.1202	kg
Equivalent spring constant	*K*_*eq*_	688.62	N/m
Equivalent damping factor	*C*_*eq*_	0.08266	Ns/m
Natural frequency	*f*_*n*_	9.17	Hz
Logarithmic decrement	*δ*	0.02172	-
Damping ratio	*ζ*	0.003457	-
Mass ratio	m*	164.5	-

After calculating the parameters mentioned in Table 2, the cylinder was subjected to airflow. The incoming air velocity (*U*_*∞*_) was varied by changing the speed of the motor which caused the Reynolds number (*Re*) to vary from 2300 to 16000. For each *Ur* value the oscillation amplitude (*A*), and voltage output (*V*) with and without resistance in the circuit were recorded using DAQ. The oscillation amplitude and frequency were recorded using an accelerometer. The results of the experimental data are plotted and discussed in Section 3.

### 2.2 Numerical method

The numerical method carried out to perform the simulation is presented in this section.

#### 2.2.1 Computational domain

**Fig 4:**
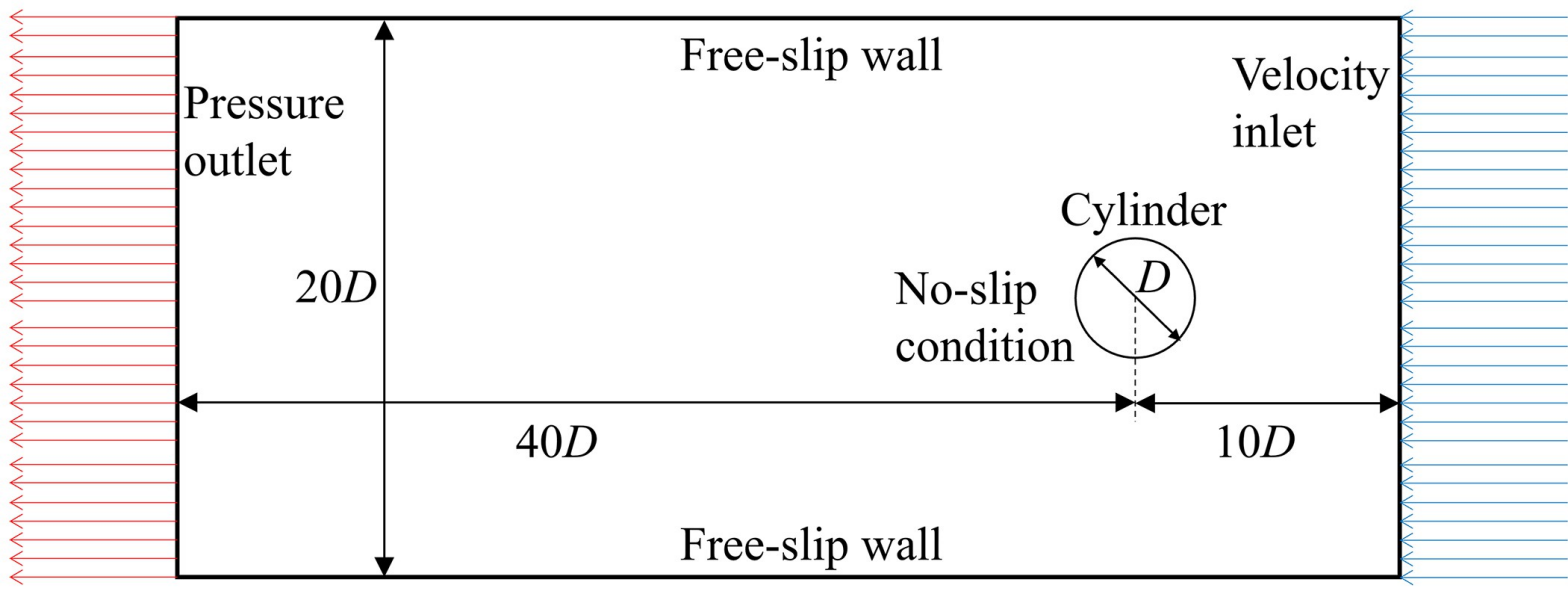
Computational domain with the prescribed boundary conditions.

The computational domain used to perform numerical simulation in Ansys/Fluent is shown in Fig 4. It consisted of a rectangular section housing a circular cylinder of diameter *D* = 50mm. The extents of the domain in streamwise (*x*) and transverse (*y*) direction were taken to be 50*D* and 20*D*, respectively, also shown in Fig 4. The transverse extents of the domain were chosen to be 20*D* to give the blockage ratio of 5%. The blockage ratio should not be higher than 5% in order to have the effects of the lateral boundaries to be negligible [41–43].

The applied boundary conditions to the walls of the domain and cylinder are also shown in Fig 4. Inlet was assigned as uniform velocity (*u = U*_*∞*_, *V =* 0), and the outlet was assigned as pressure outlet with zero gage pressure (*p*_*gage*_ = 0, *∂u/∂x =* 0, *∂V/∂x =* 0). The lateral boundaries were assigned as free-slip walls with no shear (*∂u/∂y =* 0, *V = 0*). The wall of the cylinder was considered as a no-slip wall that allowed the boundary layer to develop (*u = V =* 0). Where, *u* and *V* are velocities in *x-* and *y-* direction, respectively.

The structural parameters, which include the mass of the cylinder (*m*) and the resultant mass ratio (m^*^), equivalent spring constant (*K*_*eq*_), equivalent damping factor (*C*_*eq*_) and damping ratio (*ζ*), and the natural frequency of the structure (*f*_*n*_) were set as defined in Table 2 in order to match with the experimental values. The inlet velocity (*U*_*∞*_) was varied in the range of 0.4 to 5 m/s to vary the Reynolds number (*Re*) from 2300 to 16,000, which resulted in the variation of reduced velocity (*Ur*) from 1 to 10.

#### 2.2.2 Governing equations

Air flow over the cylinder is governed by Unsteady Reynolds-Averaged Navier-Stokes Equations (URANS). The fluid (air) was considered to be incompressible and viscous, and the properties of air were selected at the temperature of 20°C. As the Reynolds number in the analysis was higher than 2300, therefore turbulence modeling was used. The conservation of mass and momentum equations are given as:

$$\frac{\partial u_{i}}{\partial x_{i}}=0$$ (7)

$$\frac{\partial u_{i}}{\partial t}+u_{j}\frac{\partial u_{i}}{\partial x_{j}}=-\frac{1}{\rho }\frac{\partial p}{\partial x_{i}}+v\nabla^{2}u_{i}-\frac{\partial \bar{u_{i}^{\prime }u_{j}^{\prime }}}{\partial x_{j}}$$ (8)

where, *x* represents the cartesian coordinate, *u* the velocity, and *i* and *j* the directions. The pressure is represented by *p*. The Reynolds stress tensor is given as:

$$\bar{u_{i}^{\prime }u_{j}^{\prime }}=-v_{t}(\frac{\partial u_{i}}{\partial x_{j}}+\frac{\partial u_{j}}{\partial x_{i}})+\frac{2}{3}k\delta_{ij}$$ (9)

where, *v*_*t*_ is the turbulence viscosity governed by the turbulence model. *k* is the turbulence kinetic energy and is defined as *k = u*_*i*_*u*_*i*_*/2*. The Kronecker delta is expressed as *δ*_*ij*_ in the above equation. Out of the several turbulence models available, shear stress transport (SST) *k*–*ω*, developed by Menter [44] is used in this work due to its superior performance in adverse pressure and gradient flow. The two transport equations of turbulence kinetic energy (*k*), and specific dissipation rate (*ω*) are presented below:

$$\frac{\partial k}{\partial t}+\frac{\partial (ku_{i})}{\partial x_{i}}=\frac{\partial }{\partial x_{j}}[(v+\frac{v_{t}}{\sigma_{k}})\frac{\partial k}{\partial x_{j}}]-\bar{u_{i}^{\prime }u_{j}^{\prime }}\frac{\partial u_{j}}{\partial x_{i}}-\beta_{k}k\omega$$ (10A)

$$\frac{\partial \omega }{\partial t}+\frac{\partial (\omega u_{i})}{\partial x_{i}}=\frac{\partial }{\partial x_{j}}[(v+\frac{v_{t}}{\sigma_{\omega 1}})\frac{\partial \omega }{\partial x_{j}}]+\alpha S^{2}-\beta_{\omega }\omega^{2}+2(1-F_{1})\frac{1}{\sigma_{\omega 2}}\frac{1}{\omega }\frac{\partial k}{\partial x_{j}}\frac{\partial \omega }{\partial x_{j}}$$ (10B)

where, *S* and *F*_*1*_, represent the strain rate and blending function, respectively. More detail can be found in the work of Menter [44].

#### 2.2.3 Numerical technique for cylinder motion

In this paper, the “Pressure Implicit with Splitting of Operators” (PISO) algorithm was utilized to simultaneously solve the momentum and continuity equations. The unsteady terms were handled through the application of an implicit first-order scheme. For the solution of the transport equations for turbulence kinetic energy (κ) and dissipation rate (ω), a second-order upwind scheme was employed. Additionally, a second-order scheme was utilized to handle both the diffusion and convection terms. Similar numerical technique has been used by [45–47].

**Fig 5:**
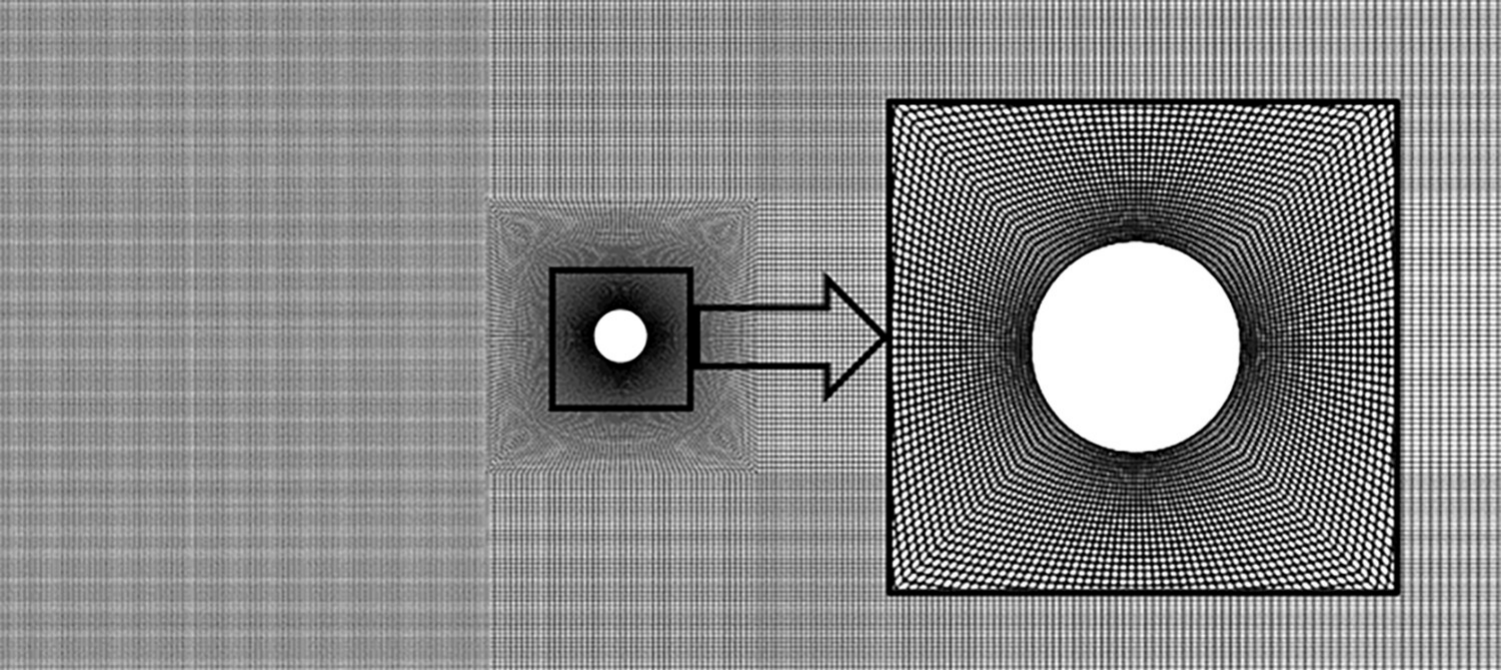
Discretized mesh with quad elements.

The cylinder motion was governed by the following equation:

$$m\ddot{y}+C_{eq}\dot{y}+K_{eq}y=F_{y}$$ (11)

where the values of *m*, *C*_*eq*_, and *K*_*eq*_ are provided in Table 2. In the above equation, $\ddot{y},\dot{y}$, and *y* represent the acceleration, velocity, and displacement of the cylinder in the transverse (*y*) direction, respectively. *F*_*y*_ is the transverse force, which was obtained from the position of the cylinder and the fluid flow, at each time instant. The dynamic mesh technique was used to allow the cylinder to vibrate. User defined function (UDF), a code written in C language, was incorporated into Fluent to account for the cylinder motion according to Eq 11. The reduced velocity (*Ur = U*_*∞*_*/f*_*n*_*D*) was varied by varying the incoming velocity (*U*_*∞*_). To ensure the solution’s accuracy, the residuals of the governing equations were set as the convergence criteria at 10^−8^. The timestep was carefully determined to meet the stringent condition of the Courant–Friedrichs–Lewy (CFL) number of ≤ 1. A well-structured mesh was established using quad elements, centered around the cylinder in the flow domain, with a total of 62,500 elements, as shown in Fig 5. This mesh was designed with a focus on the cylinder wall, including clustering near the wall to accurately account for the wall effects.

#### 2.2.4 Mesh sensitivity

Table 3 shows the mesh sensitivity analysis for the numerical study of the oscillating cylinder at *Ur* = 5. Four levels of mesh were created: coarse 1, coarse 2, baseline, and fine, with 31250, 46875, 62500, and 93750 numbers of cells, respectively. The two parameters recorded for each mesh were: root mean square (*rms*) of lift coefficient (*C*_*L*_), and dimensionless amplitude of vibration (*A/D*). The values of these parameters for each mesh were compared with those of the fine mesh and reported as a percentage error in Table 3. Compared with the fine mesh, baseline mesh performed reasonably well as the error in *rms C*_*L*_ and *A/D* was 0.59% and 1.16%, respectively. Baseline mesh was therefore chosen as a compromise between computational time and accuracy and was used for further analysis in this work.

**Table 3:** Mesh independence study for the cylinder parameters at *Ur* = 5.

Mesh	Elements	*rms C*_*L*_	Error in *rms C*_*L*_ (%)	*A/D*	Error in *A/D* (%)
Coarse 1	31,250	1.1084	3.93	0.1274	7.81
Coarse 2	46,875	1.1315	1.92	0.1328	3.91
Baseline	62,500	1.1469	0.59	0.1366	1.16
Fine	93,750	1.1537	-	0.1382	-

*Ur* = reduced velocity, *C*_*L*_ = lift coefficient, *A/D* = dimensionless amplitude of vibration

**Fig 6:**
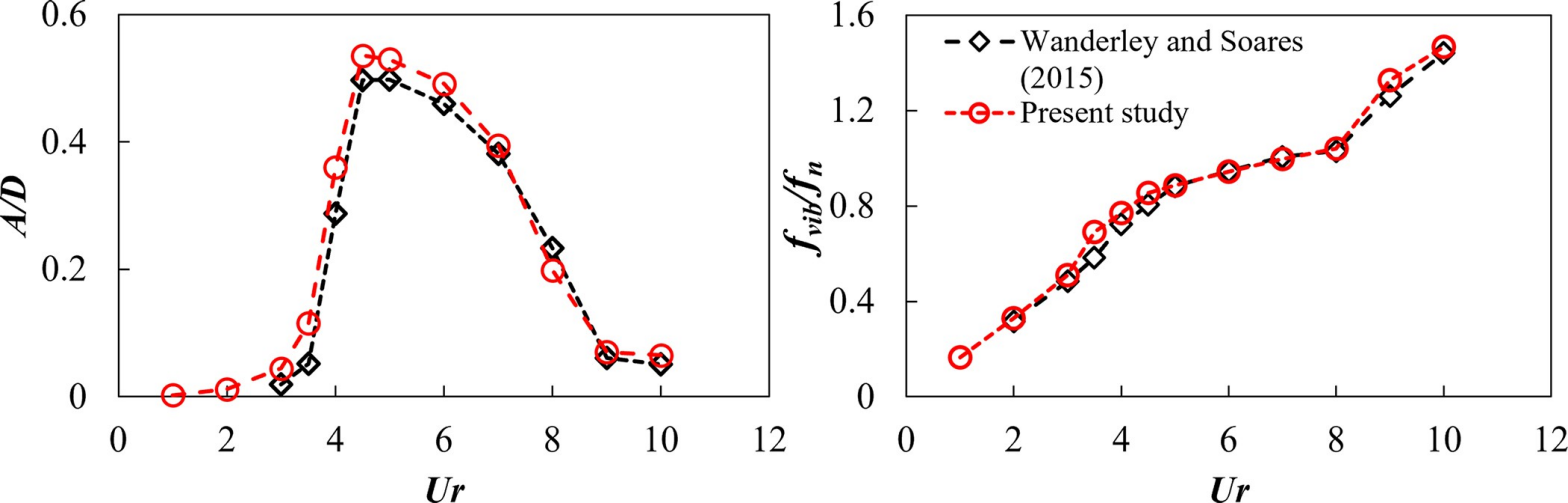
Validation of numerical model using the results of Wanderley & Soares [48].

**2.2.5 Validation of the numerical model.** The outcomes of the developed numerical model were benchmarked against the results in the literature. It was tested using a single cylinder allowed to oscillate in the transverse direction at a Reynolds number of 100, with a mass ratio of 1.88 and a damping ratio of 0.00542. The comparison focused on the dimensionless transverse amplitude of vibration (*A/D*) and the ratio of the vibration frequency to the natural frequency (*f*_*vib*_*/f*_*n*_) across different values of reduced velocities (*Ur*), referencing the work of Wanderley & Soares [48]. The results from this study closely align with those reported by Wanderley & Soares [48], as shown in Fig 6. The agreement, highlighted in Fig 6, confirms the accuracy of the numerical model, which was then used for further analysis.

## 3. Results

**Fig 7:**
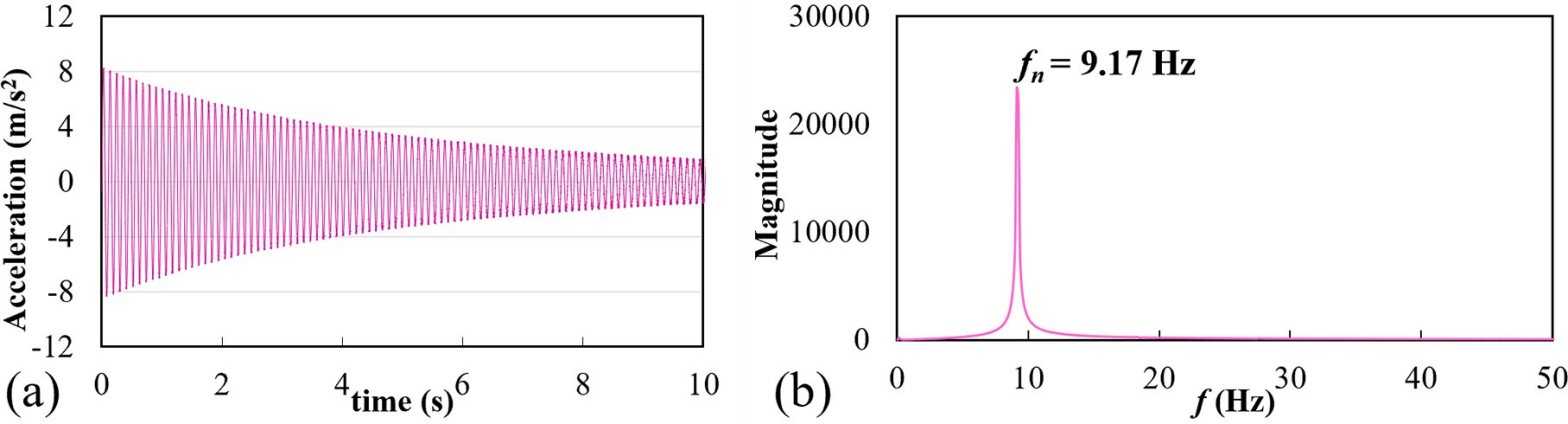
(a) Signal from the accelerometer after plucking excitation, (b) FFT of the acceleration signal showing the natural frequency of the system (*f*_*n*_).

The results obtained in this work through the experimental study and numerical analysis are discussed in this section. Prior to experimentation on wind tunnel, the natural frequency (*f*_*n*_) of the cylinder system was found out by plucking the cylinder in still air, when the wind tunnel air flow was turned off. The results in terms of acceleration signal are shown in Fig 7. The acceleration signal was picked up by the accelerometer and was recorded on the computer via NI DAQ. Taking the “Fast Fourier Transform” (FFT) of this signal, *f*_*n*_ was determined, and was found to be 9.17 Hz as shown in Fig 7.

### 3.1 Amplitude and frequency of vibration

**Fig 8:**
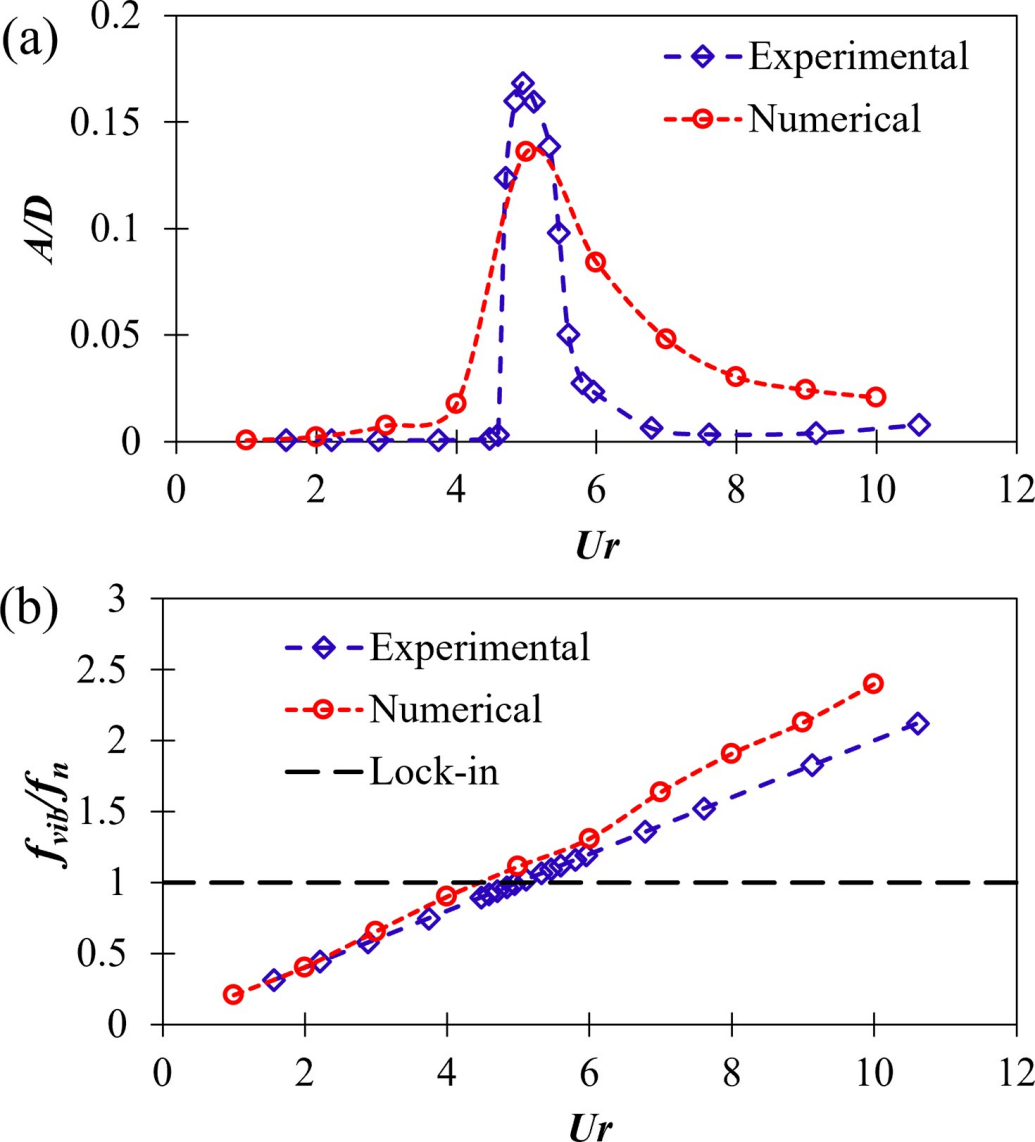
(a) Dimensionless amplitude of vibration (*A/D*), and (b) normalized frequency of vibration (*f*_*vib*_*/f*_*n*_), with varying reduced velocity (*Ur*) for experimental and numerical results.

The amplitude of vibration (*A*) is normalized with the diameter of the cylinder (*D*) and is presented as a dimensionless quantity (*A/D*) in Fig 8. Frequency of vibration (*f*_*vib*_) normalized with the natural frequency of the system (*f*_*n*_) is also presented as a dimensionless quantity (*f*_*vib*_*/f*_*n*_). The figure shows experimental as well as numerical results for direct comparison. The general trend of *A/D* versus *Ur* is similar for both experimental and numerical results, however there are some deviations. Same is true for *f*_*vib*_*/f*_*n*_ versus *Ur*.

As observed from Fig 8A, at low *Ur* the vibration amplitude is negligible for both experimental and numerical results. A big jump occurs and the peak in *A/D* is observed close to *Ur* = 5. For both experimental and numerical values, *A/D* decreases with increasing *Ur* after the peak has occurred. The highest value of *A/D* observed during experiments is 0.168, whereas that observed in numerical simulations is 0.136, leading to a difference of 19%. The possible causes of the deviation are discussed later in this section. The peak for *A/D* is observed at the values where the ratio *f*_*vib*_*/f*_*n*_ is close to 1, i.e., lock-in occurs.

From *f*_*vib*_*/f*_*n*_ versus *Ur* plot (Fig 8B), it is observed that *f*_*vib*_*/f*_*n*_ increases monotonically with *Ur*. The experimental and numerical results are found to be in good agreement with each other, with slight deviations at higher *Ur*. The region between *Ur* = 4 and 6 is the region where *f*_*vib*_ matches *f*_*n*_ and lock-in occurs. High amplitude vibrations occur in this region as observed from Fig 8A. This is the region most suitable for harvesting energy from vortex-induced vibration (VIV).

Considering the deviations in the experimental and numerical results, the differences could be due to any of the following factors or due to the combination of them:

- Experimental study is 3D, however numerical study conducted was 2D to save computational resources and to decrease computation time.
- The turbulence is modeled using SST *k*–*ω* model numerically, although this model has been used widely for similar studies [46,49–52] but it is not guaranteed that it will match with the actual turbulence phenomenon happening in the experiments.
- The blockage ratio in the experimental setup was 16.6% which is higher than the recommended value (≤ 5%) which may have caused discrepancies in the results.

Nevertheless, despite the discrepancies in the experimental and numerical results, the results are fairly close and follow the same general trend, as observed in Fig 8.

**Fig 9:**
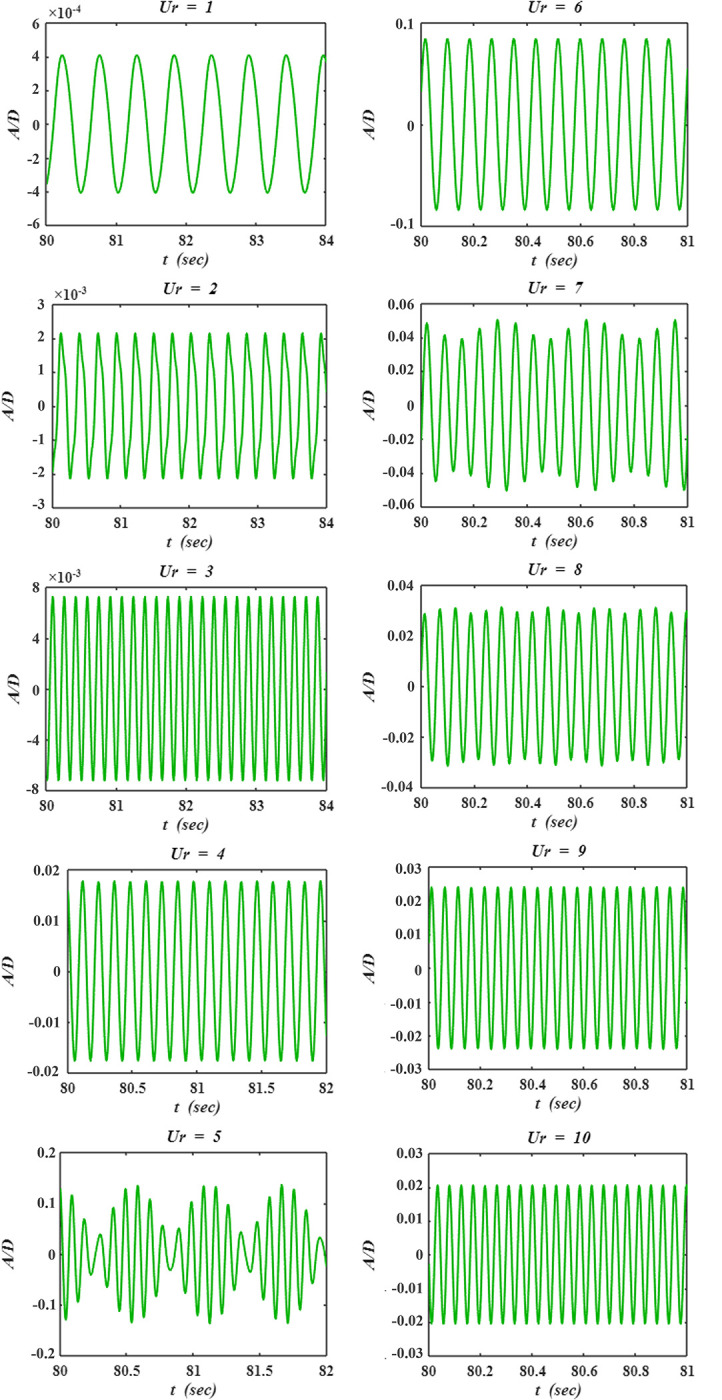
Time histories of dimensionless amplitude of vibration (*A/D*) versus flow time (*t*).

The time histories of *A/D* for a few seconds of the flow time (*t*) are presented in Fig 9. The data is recorded when the cylinder response becomes periodic. The ranges of *x-* and *y-* axes are adjusted in each sub-figure for better visualization of the signal. At low *Ur* (1–3), the amplitude of vibration is found to be negligible. *A/D* slightly increases at *Ur* = 4, and high amplitude vibrations are observed at *Ur* = 5. The trend of *A/D* versus *Ur* is more clearly visible in Fig 8A. From Fig 9, it is observed that the vibration is monotonic at most *Ur*, however modulation is observed at some of the cases, such as *Ur* = 5, 7, and 8. Modulation results due to the combined effect of the flow conditions, cylinder response, and the wake structures.

### 3.2 Lift coefficient and Strouhal number

**Fig 10:**
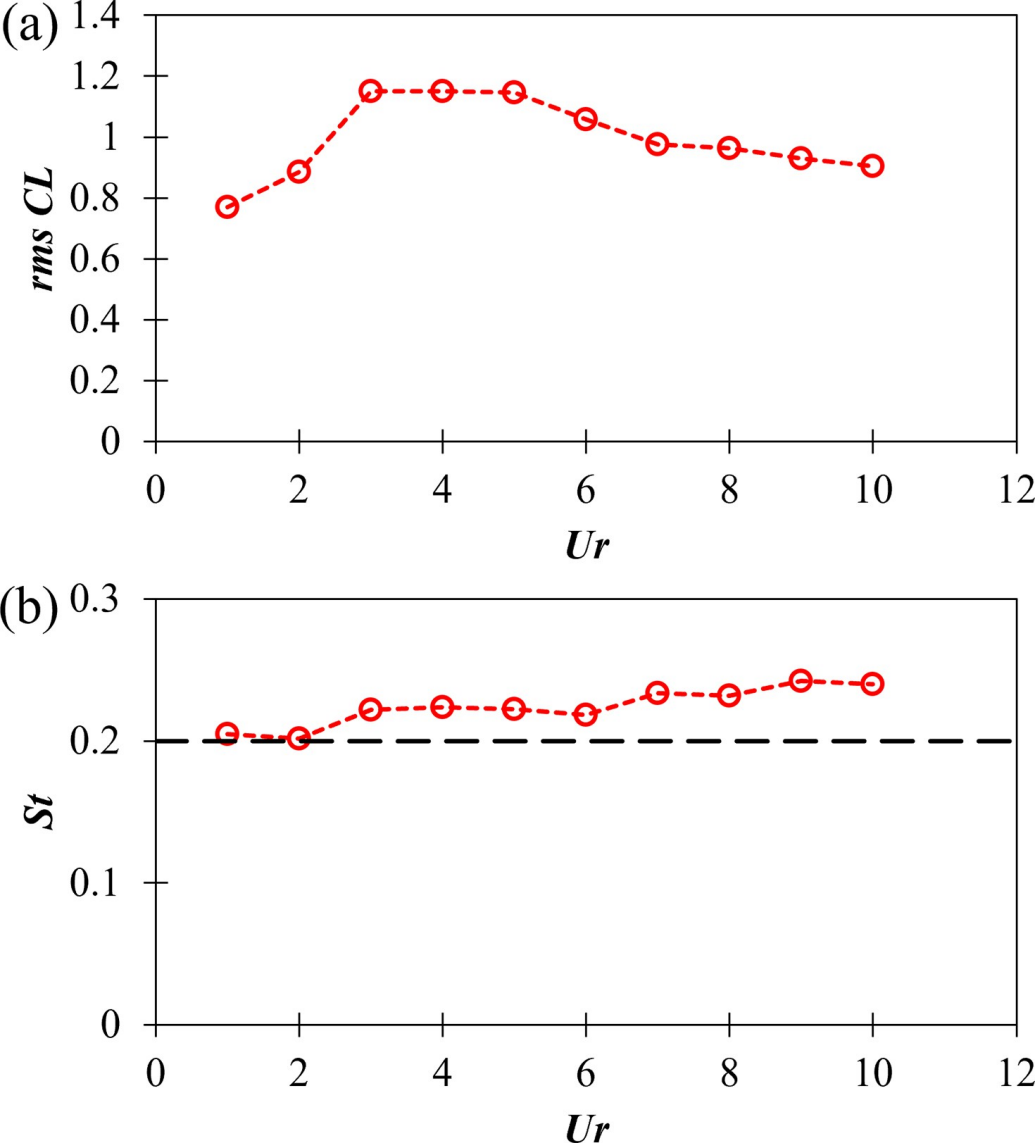
(a) Root mean square (*rms*) of lift coefficient (*C*_*L*_), and (b) Strouhal number (*St*), with varying reduced velocities (*Ur*).

**Fig 11:**
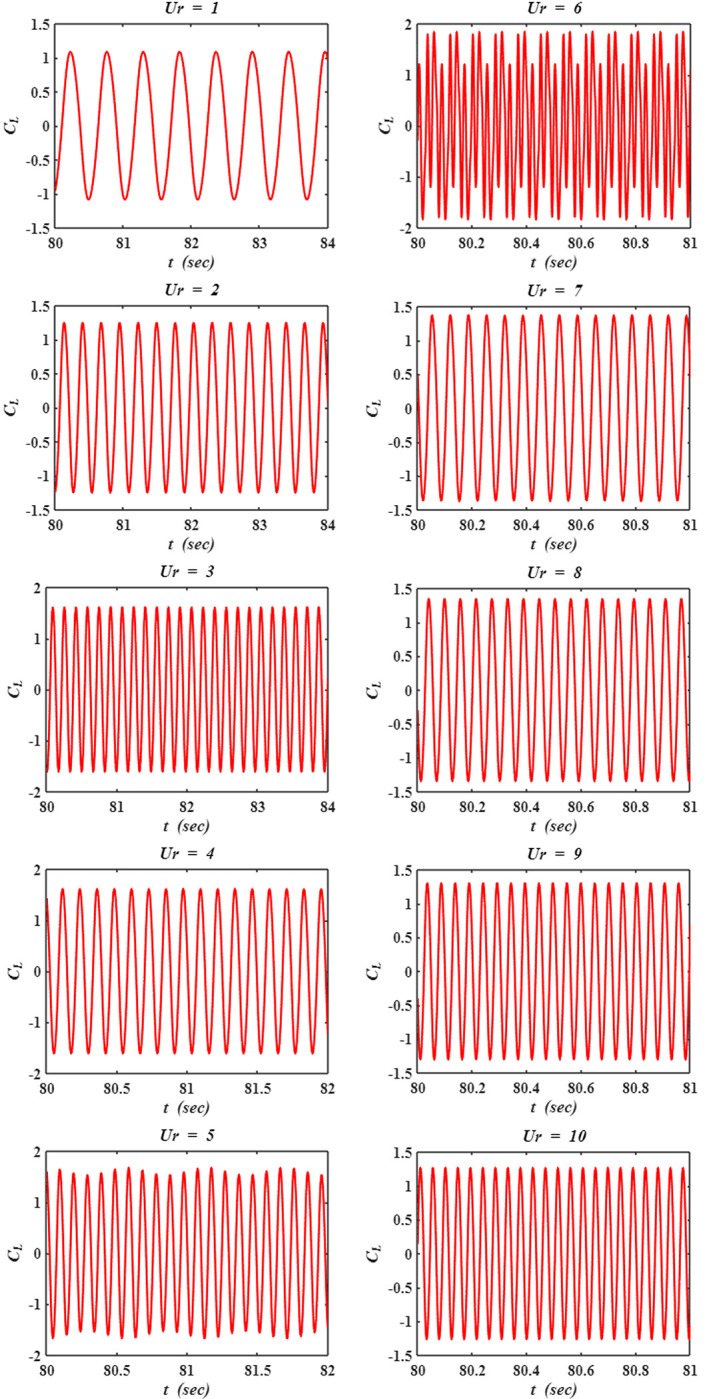
Time histories of the lift coefficient (*C*_*L*_) versus flow time (*t*).

The behavior of the root mean square (*rms*) of lift coefficient (*C*_*L*_) and Strouhal number (*St*) is shown in Fig 10 with varying reduced velocity (*Ur*). Strouhal number is considered as the dimensionless vortex shedding frequency, and is expressed as *St = f*_*vs*_.*D/U*_*∞*_, where *f*_*vs*_ is the vortex shedding frequency. For *C*_*L*_, *rms* is chosen as the metric over average value as *C*_*L*_ fluctuates about zero average as shown in Fig 11. From Fig 10A it is observed that *rms C*_*L*_ increases with increasing *Ur* from *Ur* = 1 to 3 and stays constant until *Ur* = 5. Further on it decreases with increasing *Ur*.

From Fig 10B, it is observed that the Strouhal number (*St*) stays close to 0.2 at all values of reduced velocity in the range of *Ur* = 1–10. It is well known that in the subcritical regime (300 < *Re* < (1.5–2)×10^5^), for the flow over a stationary cylinder, the Strouhal number stays constant at *St* ≈ 0.2 [53–56]. The value of *St* = 0.2 is shown as a broken black line in Fig 10B for reference. A slight increasing trend is observed for *St* in Fig 10B with increasing *Ur*, meaning that the vortices shed slightly faster at higher *Ur* as compared to low *Ur*.

The time histories of *C*_*L*_ at each *Ur* are shown in Fig 11 with the flow time (*t*) varying for a few seconds. Similar to Fig 9, the time histories of *C*_*L*_ in Fig 11 are recorded when the response of the cylinder becomes periodic. At most of the cases the lift signal is found to be monotonic, however modulation is observed in few cases (*Ur* = 5 and 6).

### 3.3 Vortex shedding

**Fig 12:**
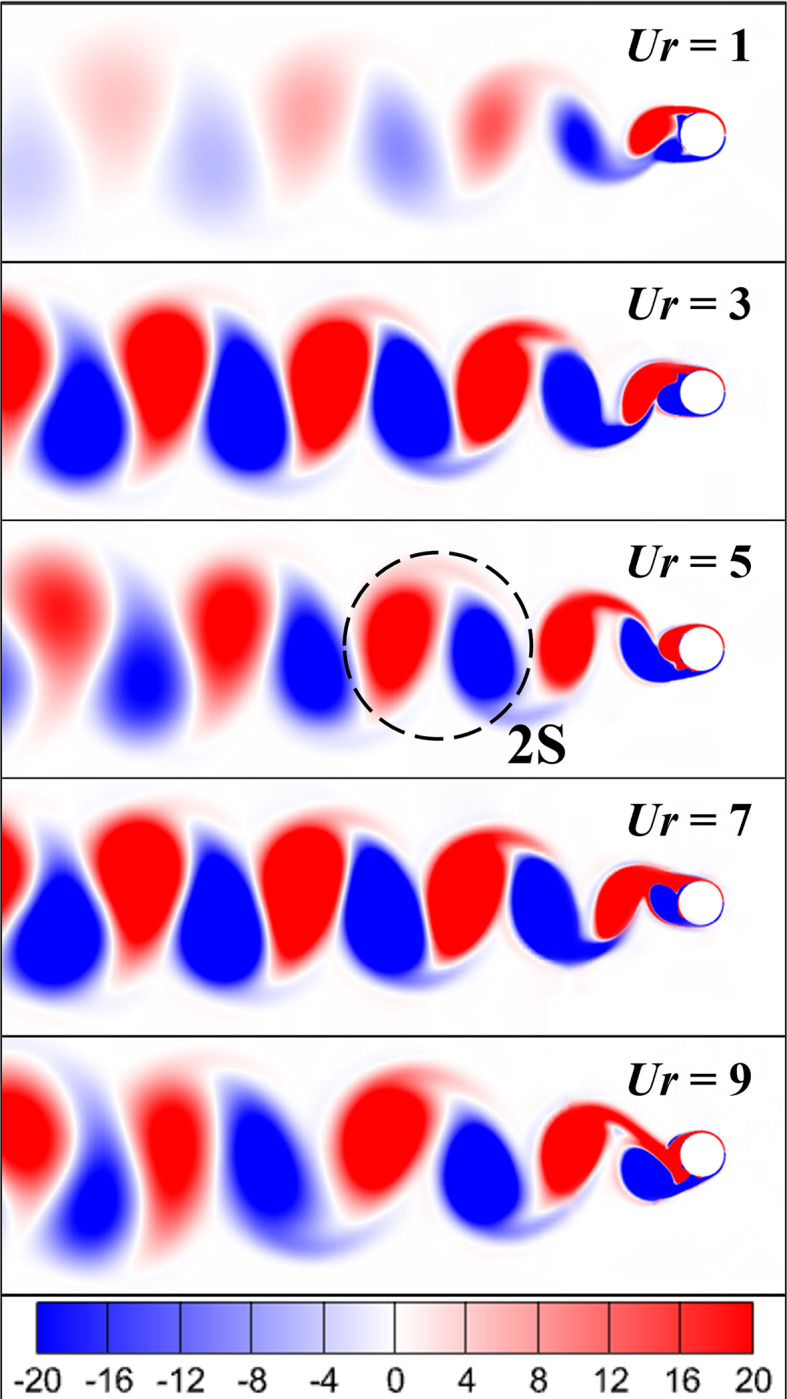
Vorticity contours showing vortex shedding patterns for selected cases.

The vorticity contours showing the shedding of vortices and the vorticity pattern are displayed in Fig 12. The viscous flow over the cylinder coupled with no-slip condition on the surface of the cylinder leads to boundary layer development. As the fluid keeps on flowing, the shear layers are stretched leading to the flow separation, and eventually roll up in the wake of the cylinder to form vortices. The periodic shedding of vortices induces the aerodynamic forces in the cylinder leading to flow-induced vibrations, or more specifically vortex-induced vibrations.

In Fig 12, the 2S vortex shedding pattern is observed in all cases where two single vortices are shed in one complete cycle, i.e., one single vortex from each side (top and bottom) of the cylinder [57–59]. Since the vortex shedding pattern is similar, therefore the contours are plotted for selected cases only (*Ur* = 1, 3, 5, 7, 9). Weak 2S pattern is observed at *Ur* = 1, however at *Ur* = 3, 5, 7, and 9, strong 2S pattern is observed.

### 3.4 Energy harvesting

**Fig 13:**
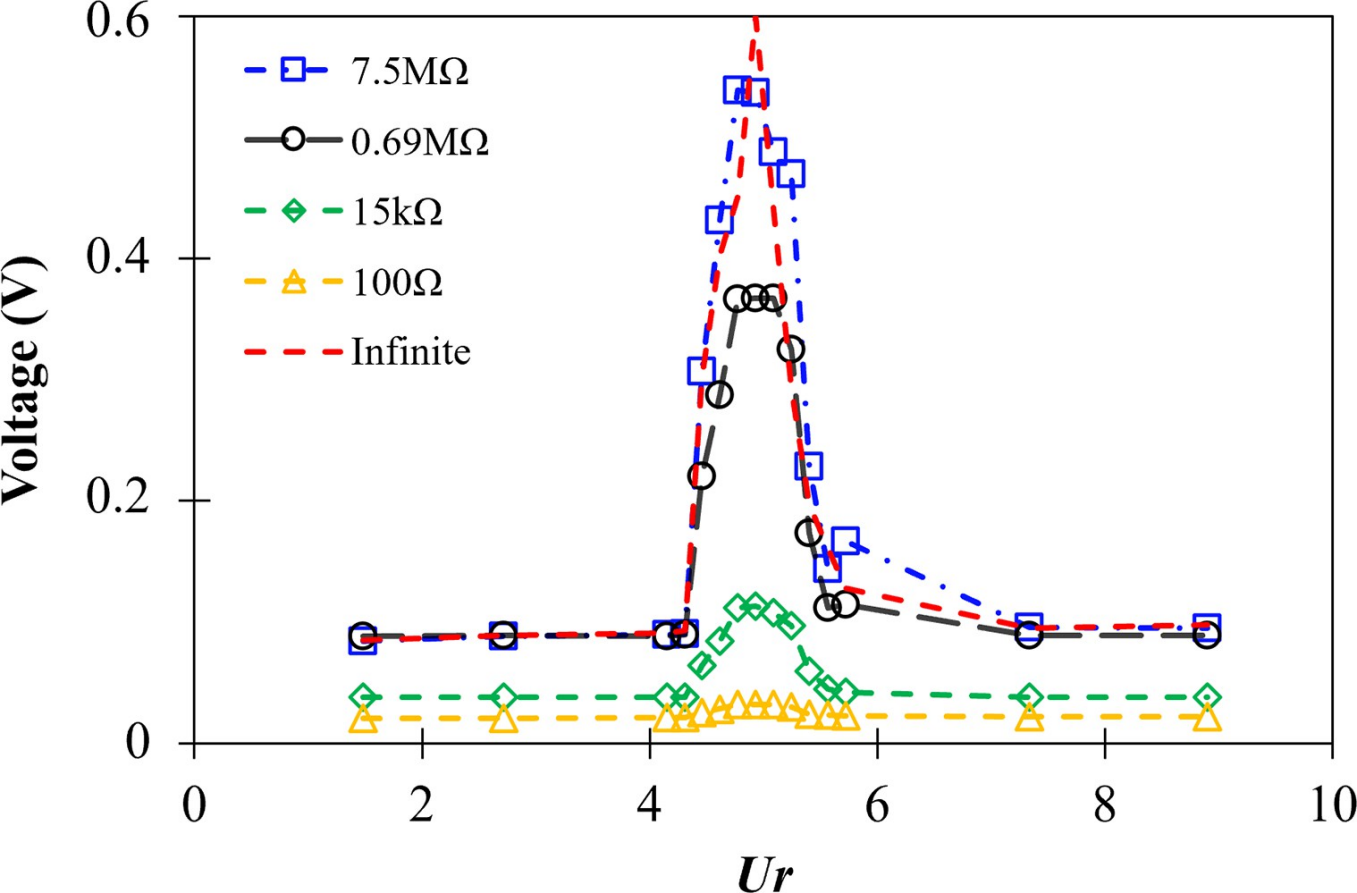
Output voltage measured across the piezoelectric using various resistance values.

The output voltage from the piezoelectric generator is presented in Fig 13 with varying reduced velocities (*Ur*). The resistances used to measure the output voltage ranged from low resistance (100Ω) to high resistance (7.5MΩ). The circuit diagram shown in Fig 3 was used to measure the output voltage across the MEMS.

Generally, it is observed that the output voltage increases with increasing resistance value. The voltage output follows the trend of the *A/D* observed in Fig 8A. The maximum output voltage measured across each resistance is observed in the range of *Ur* = 4.5–5.5, where maximum *A/D* was observed. In this region lock-in occurs and the shedding frequency synchronizes with the structure’s natural frequency (Fig 8B). In the lock-in region, the intensity of oscillations increases, leading to higher deflection of the MEMS, which generates higher voltage output. Therefore, the region of interest is between *Ur* = 4 and 6 that gives the maximum output voltage.

It is also noticed that the output voltage and the nondimensional amplitude of vibration are inline, i.e., the higher *A/D* gives a higher output voltage. The maximum *A/D* of 0.168 was observed at *Ur* = 4.95, and the maximum output voltage at this condition with infinite resistance (open circuit) is observed to be 0.6V. The output voltage of the MEMS depends on the resistance value of the circuit. It is observed from Fig 13 that as the resistance value is reduced, the output voltage peak drops.

**Fig 14:**
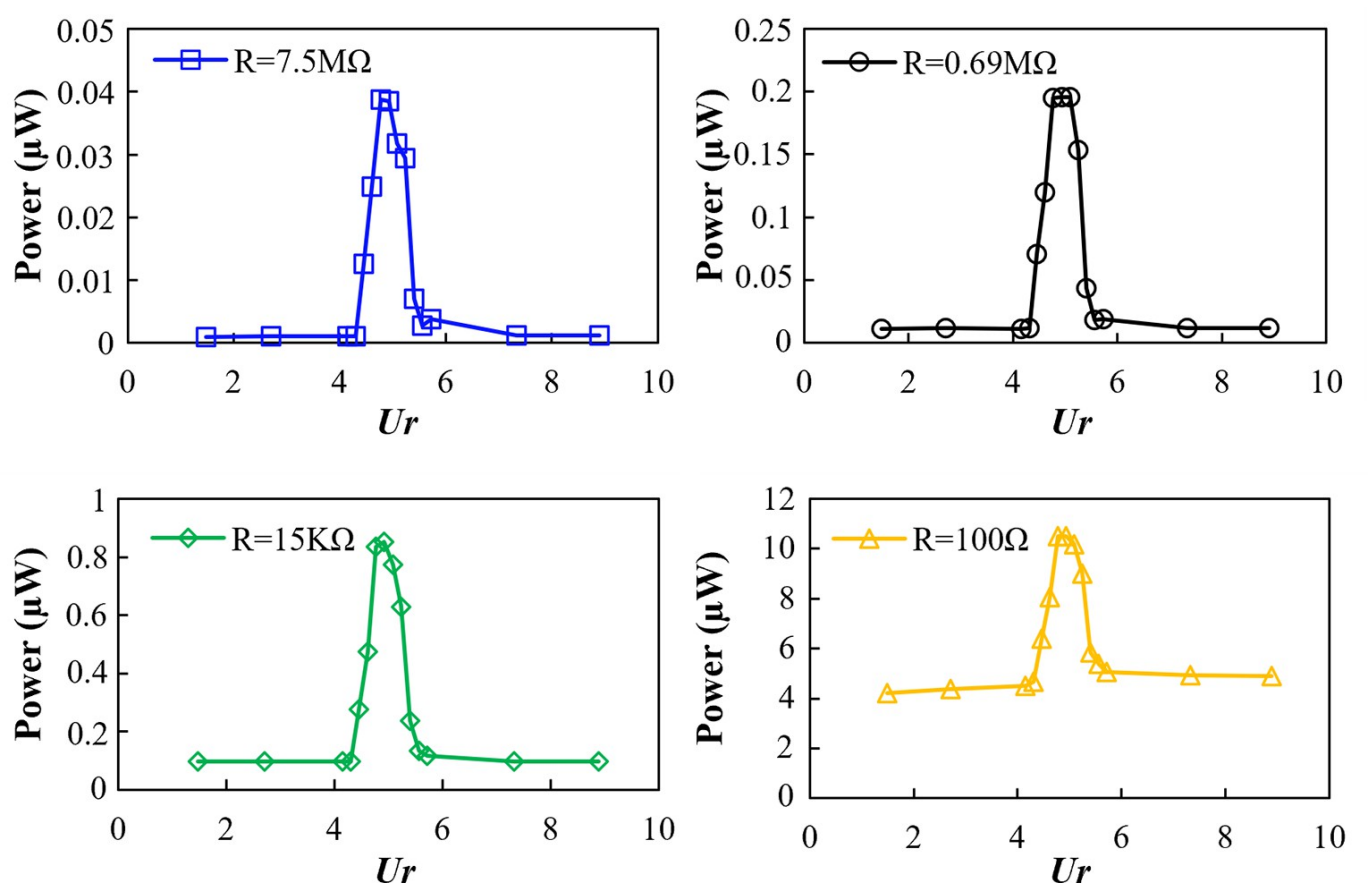
Power output with different resistance values.

The recorded voltage is converted to the power output of the MEMS using the mathematical relation *P = V*^*2*^*/R*, where *P* is the output electrical power, *V* is the output voltage, and *R* is the resistance value. Fig 14 shows the plots of the power output recorded against the *Ur* for different values of circuit resistance. The maximum output power of 10.5 μW is recorded for 100Ω resistance at *Ur* = 4.95, whereas the maximum output power with 7.5MΩ turned out to be a mere 0.04μW. Generally, as the resistance value increases, the power output decreases.

While the output power currently remains at a low level, this study highlights the significant potential for energy harvesting from FIV. Enhancements to the system’s performance can be achieved by optimizing its structure, particularly through the utilization of a system with a lower mass-damping ratio (m*ζ). Additionally, the shape of the cylinder can be fine-tuned. These adjustments can result in a higher vibrational amplitude, leading to more substantial deflection in the MEMS. Despite the current limitations, it is worth noting that fluid energy is abundantly available in the form of wind, wave energy, and ocean and river currents. Installing multiple units, similar to the proposed setup, has the potential to harness this energy and produce useful amounts of power.

## 4. Conclusion

This study aimed to investigate the potential of flow-induced vibration (FIV) as a source of sustainable and renewable energy. The abundant energy found in wind and ocean currents can be tapped by deploying numerous installations similar to the suggested model to produce useful power. The experimental setup consisted of a 50mm diameter cylinder mounted elastically with the help of helical and leaf springs. The Reynolds number varied from 2300 to 16000 depending on the incoming air flow velocity. The cylinder was allowed to oscillate only in the transverse direction, as the transverse vibrations are more significant than their counterpart the streamwise vibration. The numerical study complemented the experimental results, showing a good agreement with a few discrepancies. SST *k–ω* turbulence model was used to model the turbulence numerically. Dynamic mesh technique was deployed with user-defined-function to account for cylinder motion in Ansys/Fluent. The results revealed that the Strouhal number remained fairly close to the expected value of 0.2, and the vorticity contours showed 2S vortex shedding pattern. The maximum dimensionless amplitude of vibration (*A/D*) obtained was 0.168 and 0.136 experimentally and numerically, respectively. To harvest the mechanical energy of the vibrating cylinder, a micro-electromechanical system (MEMS), which is a piezoelectric bending transducer, was mounted on the leaf spring. The results revealed that high vibrational amplitudes occurred in the lock-in region (*Ur* = 4.5–6), with the peak occurring at *Ur* = 4.95. Maximum voltage and power output were also observed when the maximum amplitude occurred. Increasing the circuit resistance increased the output voltage, but it decreased the output power. The maximum output voltage of 0.6 V and the maximum power of 10.5 μW were recorded under specific conditions, indicating the potential of this energy source to generate power. The experimental and numerical analyses emphasized the importance of the lock-in region, where maximum amplitude of vibration is observed, resulting in maximum power output. In conclusion, the findings of this study suggest that FIV can be harnessed as a clean and renewable source of energy, and further research could explore ways to optimize the design of FIV-based energy systems for practical applications.

## Supporting information

S1 Table Data for figures. (XLSX)

## Abbreviations

*A* m: Amplitude of oscillation
*A/D* -: Dimensionless amplitude of vibration
*C*_*eq*_ N-s/m: Equivalent damping factor
*C*_*L*_ -: Lift coefficient
*D* m: Dimeter of the cylinder
*D*_*X*_ m: Diameter of the cross-section of the wind tunnel test section
*f*_*n*_ Hz: Structural natural frequency
*f*_*vib*_ Hz: Frequency of vibration
*f*_*vs*_ Hz: Vortex shedding frequency
*F*_*y*_ N: Force exerted on the cylinder in *y*-direction
*k* m^2^/s^2^: Turbulence kinetic energy
*K*_*eq*_ N/m: Equivalent spring constant
*L×W×H* m: Length × Width × Height of the wind tunnel test section
*l×w×h* m: Length × Width × Height of the piezoelectric strip
*m* kg: Mass of cylinder
*m** -: Mass ratio
*P* W: Electrical power output
R Ω: Resistance
*Re* -: Reynolds number
*rms* -: Root mean square
St -: Strouhal number
*t* s: Time
*Ur* -: Reduced velocity
*U*_*∞*_ m/s: Freestream velocity
*u* m/s: Velocity component in *x*-direction
*V* m/s: Velocity component in *y*-direction
*v*_*t*_ m^2^/s: Turbulent viscosity
*V* V: Voltage
*y* m: Transverse displacement
*ẏ* m/s: Transverse velocity
*ÿ* m/s^2^: Transverse acceleration
δ -: Logarithmic decrement
δ_ij_ -: Kronecker delta
*ρ* kg/m^3^: Density of the fluid
*μ* N.s/m^2^: Dynamic viscosity of the fluid
*ω* m^2^/s^3^: Dissipation rate of turbulence energy
*ζ* -: Damping ratio
